# Interstitial Lung Disease and Risk of Lung Cancer

**DOI:** 10.1001/jamanetworkopen.2025.19630

**Published:** 2025-07-09

**Authors:** Hui Xu, Li Yin, Weiwei Bian, Mingqiang Kang, Hans-Olov Adami, Weimin Ye

**Affiliations:** 1 Department of Medical Epidemiology and Biostatistics, Karolinska Institutet, Stockholm, Sweden; 2 Department of Thoracic Surgery, Fujian Medical University Union Hospital, Fuzhou, China; 3 Ming Wai Lau Centre for Reparative Medicine, Karolinska Institutet, Hong Kong, China; 4 Department of Cardiothoracic Surgery, Affiliated Hospital of Putian University, Putian, China; 5 Clinical Effectiveness Research Group, Institute of Health and Society, University of Oslo, Oslo, Norway; 6 Department of Epidemiology, Harvard T.H. Chan School of Public Health, Boston, Massachusetts; 7 Department of Epidemiology and Health Statistics, School of Public Health, Fujian Medical University, Fuzhou, China

## Abstract

**Importance:**

An association between interstitial lung disease (ILD) and lung cancer has been hypothesized but never established. To date, whether this potential association persists after controlling for genetic factors has not been addressed.

**Objective:**

To evaluate the association between ILD and the subsequent risk of different histological subtypes of lung cancer in the general population and a sibling-controlled cohort.

**Design, Setting, and Participants:**

This cohort study incorporated both population-based and sibling-controlled designs and used data from the Swedish Total Population Register and Swedish Multi-generation Register. Participants included individuals born between 1932 and 1987, with follow-up starting January 1, 1987, and continuing until December 31, 2016. Data were analyzed from February 15, 2024, to January 30, 2025.

**Exposures:**

ILD.

**Main Outcomes and Measures:**

The association between ILD and lung cancer was evaluated using multivariable hazard ratios (HRs) and 95% CIs.

**Results:**

Among the 5 425 976 individuals involved in this study (14 624 with ILD and 5 411 352 from the general population), 2 779 108 (51.2%) were male and most (2 068 062 [38.1%]) were 20 to 40 years of age. Among patients with ILD, most (9630 [65.9%]) were older than 40 years. During a 30-year follow-up period, 40 592 cases of lung cancer were diagnosed among those without ILD (incidence rate, 26.2 per 100 000 person-years) and 227 cases among those with ILD (incidence rate, 355.4 per 100 000 person-years). After adjusting for sex, age, calendar period, educational attainment, and smoking-related diseases, individuals with ILD had a higher risk of lung cancer (HR, 2.16; 95% CI, 1.89-2.46). Sibling-controlled analyses showed a higher risk (HR, 2.91; 95% CI, 1.98-4.27). Elevated risks were observed for adenocarcinoma (HR, 1.60; 95% CI, 1.28-2.01), squamous cell carcinoma (HR, 2.56; 95% CI, 1.99-3.29), small cell carcinoma (HR, 3.29; 95% CI, 2.32-4.68), and other histological types (HR, 2.32; 95% CI, 1.78-3.01). Results from sibling-controlled analyses generally mirrored these findings.

**Conclusions and Relevance:**

This large prospective cohort study found that ILD was associated with increased risk of most histological subtypes of lung cancer. Therefore, the presence of ILD should be included in lung cancer risk assessment models.

## Key Points

**Question:**

After controlling for genetic factors, is interstitial lung disease (ILD) still associated with an increased risk of any subtypes of lung cancer?

**Findings:**

In this cohort study of 5 425 976 participants with a sibling-controlled design to account for genetic factors, ILD was independently associated with higher risk of lung cancer. This association was observed across all histological subtypes of lung cancer.

**Meaning:**

These findings suggest that ILD should be incorporated into lung cancer risk assessment models.

## Teaser

This cohort study incorporates population-based and sibling-controlled designs with an extended follow-up to investigate the association of interstitial lung disease with lung cancer subtypes while controlling for genetic bias among the population of Sweden.

## Introduction

Lung cancer, one of the most common malignant tumors in Sweden and worldwide,^1,2^ is primarily attributed to tobacco smoking as the leading risk factor.^3^ The risk of lung cancer decreases after smoking cessation.^4^ However, current research also indicates that the decline in lung cancer incidence following smoking cessation is not as pronounced as expected, with even a sustained increase observed in men aged 70 to 84 years.^5^

Interstitial lung disease (ILD), a progressive chronic lung condition, can be caused by various environmental toxins, autoimmune diseases, infections, and genetic factors.^6,7^ During the past few decades, there has been ongoing discussion regarding the association between ILD and the occurrence of lung cancer.^8^ Some studies show an increased incidence of lung cancer among patients with ILD,^9,10,11^ while 2 studies reported a reduced incidence.^12,13^ Genetic factors are considered to play crucial roles in the pathogenesis of both ILD^3^ and lung cancer,^7^ and certain germline mutations may occur concurrently in idiopathic pulmonary fibrosis (IPF) and lung cancer.^14,15^ However, no study has comprehensively analyzed the risk of lung cancer in patients with ILD after controlling for genetic factors. Furthermore, the limited sample sizes in previous studies impeded comprehensive analysis of the various histological subtypes of lung cancer. Hence, we conducted a large-scale cohort study incorporating both population-based and sibling-controlled designs with an extended follow-up, enhancing the capacity to investigate the association of ILD with lung cancer subtypes while controlling for genetic bias.

## Methods

### Study Design

**Figure 1.**
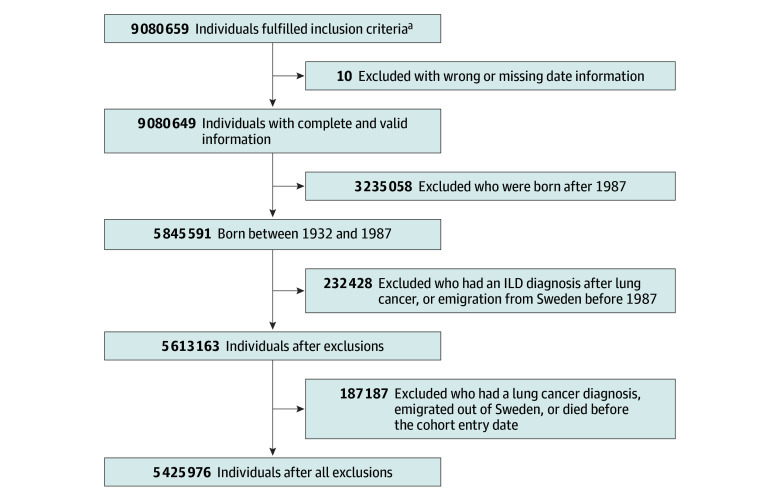
Flowchart of Patient Selection Process ILD indicates interstitial lung disease. ^a^Includes all individuals born between 1932 and 2016 whose parents were also born in Sweden.

The Swedish Total Population Register contains data on births, deaths, family relationships, and migration, enabling the identification of general population controls in cohort studies.^16^ We conducted a cohort study including all individuals born between 1932 and 1987 whose parents were also born in Sweden, using data from the Total Population Register. Study participants were further linked to the Swedish Cancer Register^17^ and Cause of Death Register^18^ by use of their individually unique national registration numbers.^19^ Each participant was followed up from January 1, 1987, until the first diagnosis of lung cancer, emigration from Sweden, death, or December 31, 2016, whichever occurred first. Participants who had a lung cancer diagnosis, emigrated from Sweden, or died before the cohort entry date were excluded from the study. The detailed selection process is shown in Figure 1. The study was approved by the Swedish Ethical Review Authority, which did not require informed consent for the use of registry data. This study followed the Strengthening the Reporting of Observational Studies in Epidemiology (STROBE) guideline.

Our final cohort included 5 425 976 individuals. Using the Total Population Register, Statistics Sweden produces the Multi-generation Register, with nearly complete familial information for individuals born since 1932.^20^ In this study, we conducted a sibling comparison by identifying full siblings through the Multi-generation Register. We included 9157 participants from the cohort who had a diagnosis of ILD and at least 1 full sibling, along with 21 725 full siblings without ILD. This study was designed to minimize familial confounding by genetic and early environmental factors.

### Ascertainment of ILD

The Swedish National Patient Register was established to systematically gather data on all inpatient care provided within the public health care system of Sweden. Initially starting in specific regions, its coverage gradually expanded until full implementation was achieved by 1987. Since 2001, outpatient care has also been included.^21^ We identified ILD using the recorded diagnoses from the National Patient Register with codes from the *International Classification of Diseases, Seventh Revision* (*ICD-7*) (524.99), *ICD-8* (517.01, 519.21, and 519.22), *ICD-9* (516), and *International Statistical Classification of Diseases and Related Health Problems, Tenth Revision* (*ICD-10*) (J84).

### Ascertainment of Outcome

Since 1958, newly diagnosed cancers have been required to be reported to the Swedish Cancer Register,^17^ ensuring nationwide comprehensive data collection on cancer incidence. The main outcome of our study was overall lung cancer, identified using *ICD-7* code 1621 in the Swedish Cancer Register. We subsequently identified histological subtypes of lung cancer using histopathological diagnoses classified according to a 3-digit coding system within the Swedish Cancer Register. For more detailed histological classification, we defined the subtypes based on the *International Classification of Diseases for Oncology, Second Edition*. Detailed code information is provided in eTable 1 in Supplement 1.

### Covariates

We obtained data on sex and age from the Total Population Register. As a proxy for socioeconomic status, we used information on the highest educational level attained (0-9, 10-12, or >12 years or missing), which was obtained from the Longitudinal Integration Database for Health Insurance and Labour Market Studies.^22^ Diagnoses of tobacco use (*ICD-8* code 989.90; *ICD-9* code 305B; or *ICD-10* codes F17, T65.2, Z71.6, Z72.0, and Z864A) and chronic obstructive pulmonary disease (*ICD-7* code 502; *ICD-8* codes 491 and 492; *ICD-9* codes 491 and 492; or *ICD-10* codes J41 to J44), retrieved from the National Patient Register, were used as a proxy for smoking history. These diagnoses were combined into a single variable termed *smoking-related diseases*.

### Statistical Analysis

Data were analyzed from February 15, 2024, to January 30, 2025. We calculated the crude incidence rate (IR) of lung cancer among individuals with (exposed) and without (unexposed) a diagnosis of ILD, dividing the number of incident cancer cases by the accumulated person-years. Exposure status was treated as a time-varying variable. Individuals with a diagnosis of ILD before cohort entry contributed all follow-up time to the exposed group, while those diagnosed during follow-up contributed time to the unexposed group before the first diagnosis and to the exposed group after the first diagnosis. If an individual received no ILD diagnosis, they contributed all person-time to the unexposed group. To alleviate concerns about surveillance bias or reverse causation, the first year of follow-up and incident outcomes were excluded from the exposed group and included in the unexposed group.

We fitted Cox proportional hazards models to estimate the mean hazard ratio (HR) and its corresponding 95% CI for overall lung cancer and various histological subtypes associated with exposure. Attained age was the underlying time scale, with adjustments for sex, educational level, calendar period at follow-up, and smoking-related diseases. In the sibling comparison, family identifiers were further used as strata in conditional Cox proportional hazards models. To assess potential effect modifications by selected factors, we also performed stratified analyses by sex, age at cohort entry, and educational attainment for overall lung cancer. We used a χ^2^ test to examine the statistical significance of the difference between the HRs of different subgroups.

Finally, we conducted a series of sensitivity analyses. First, to mitigate the potential confounding by smoking on all types of lung cancers, we excluded all participants with a history of smoking-related diseases from our study cohort. To assess the potential impact of unmeasured confounding, we calculated the E value. The E value represents the minimum strength of association that an unmeasured confounder would need to have with both the exposure and the outcome, conditional on the measured covariates, to fully account for the observed association. A larger E value suggests that only a strong unmeasured confounder could explain away the observed effect, whereas a smaller E value indicates that even a weak confounder might suffice.^23^ In the main analysis, we reassigned the first year of follow-up from the exposed group to the unexposed group. To further diminish any surveillance bias, we subsequently extended our exclusion period to encompass the first 3 years of follow-up. Last, although the completeness of the Swedish Cancer Register exceeds 98%, cases without pathological or cytological confirmation are underreported. Therefore, we further included lung cancer records from the Cause of Death Register as an outcome in our sensitivity analyses.

Data management and analyses were performed using SAS, version 9.4 (SAS Institute Inc), and R, version 4.3.3 (R Program for Statistical Computing). A 2-sided *P* < .05 was considered statistically significant.

## Results

This study included 5 425 976 individuals, of whom 2 779 108 (51.2%) were male, 2 646 868 (48.8%) were female, and most (2 068 062 [38.1%]) were 20 to 40 years of age. Participants included 5 411 352 from the general population and 14 624 who had ILD at baseline or developed ILD during follow-up. Notably, ILD was more common among male participants (8495 [58.1%]), and most patients with ILD (9630 [65.9%]) were older than 40 years. We identified 9157 individuals with ILD and having at least 1 full sibling without ILD. In the sibling cohort, 5286 participants (57.7%) were male (Table 1).

**Table 1.** Baseline Characteristics of Study Populations

Characteristics	No. (%) of participants			
	Population comparison		Sibling comparison	
	Individuals with ILD (n = 14 624)	General population (n = 5 411 352)	Individuals with ILD (n = 9157)	Siblings without ILD (n = 21 725)
Sex				
Male	8495 (58.1)	2 770 613 (51.2)	5286 (57.7)	11 030 (50.8)
Female	6129 (41.9)	2 640 739 (48.8)	3871 (42.3)	10 695 (49.2)
Age at cohort entry, y				
<20	876 (6.0)	2 043 601 (37.8)	638 (7.0)	1308 (6.0)
21-40	4118 (28.2)	2 063 944 (38.1)	3084 (33.7)	9036 (41.6)
>40	9630 (65.9)	1 303 807 (24.1)	5435 (59.4)	11 381 (52.4)
Educational attainment, y				
0-9	5337 (36.5)	1 030 406 (19.0)	3196 (34.9)	7785 (35.8)
10-12	6238 (42.7)	2 413 434 (44.6)	4003 (43.7)	9325 (42.9)
>12	3016 (20.6)	1 921 915 (35.5)	1951 (21.3)	4562 (21.0)
Missing	33 (0.2)	45 597 (0.8)	7 (0.1)	53 (0.2)

Abbreviation: ILD, interstitial lung disease.

During follow-up, 40 592 cases of lung cancer were observed among individuals without ILD (IR, 26.2 per 100 000 person-years) and 227 among patients with ILD (IR, 355.4 per 100 000 person-years). In the population comparison, individuals with ILD had a higher risk of lung cancer (HR, 2.16; 95% CI, 1.89-2.46), compared with individuals without ILD after adjustment for sex, age, and calendar period at follow-up, highest educational attainment, and smoking-related diseases (Table 2). In the sibling comparison, we also found an increased risk of lung cancer in patients with ILD (HR, 2.91; 95% CI, 1.98-4.27). In sensitivity analyses, exclusion of the first 3 years of follow-up among patients with ILD did not substantially alter the results (HR, 2.02; 95% CI, 1.71-2.37) (eTable 2 in Supplement 1). When the analysis was restricted to individuals without smoking-related diseases—used as a proxy for smoking exposure—the increased risk of lung cancer among patients with ILD remained evident (HR, 3.68; 95% CI, 3.07-4.41) (eTable 3 in Supplement 1). We also calculated the E value (4.41) for the association to assess the potential role of unmeasured confounding factors in the exposure-outcome association.^23^ Given that the smoking-related risk for ILD ranges from 1.6 to 2.3,^24^ which is lower than the E value, we concluded that residual confounding by smoking alone is insufficient to fully explain the observed association. In addition, we calculated the raw counts and IRs based on the cross-tabulation of smoking-related diseases and exposures. The results indicated that individuals with both smoking-related diseases and ILD exhibited a higher IR than those with either condition alone (eTable 4 in Supplement 1).

**Table 2.** IR and HR for Association of Lung Cancer With ILD (1-Year Lag Time)

Diagnosis with ILD	Population comparison		Sibling comparison	
	No. of cases with lung cancer/IR per 100 000 person-years	HR (95% CI)^a^	No. of cases with lung cancer /IR per 100 000 person-years	HR (95% CI)^b^
No	40 592/26.2	1 [Reference]	682/82.4	1 [Reference]
Yes	227/355.4	2.16 (1.89-2.46)	145/343.1	2.91 (1.98-4.27)

Abbreviations: HR, hazard ratio; ILD, interstitial lung disease; IR, incidence rate.

^a^ Adjusted for attained age, sex, smoking-related diseases, calendar period at follow-up, and highest educational attainment.

^b^ Adjusted for attained age, sex, smoking-related diseases, calendar period at follow-up, and highest educational attainment and stratified by family identifiers.

As shown in Table 3, the risk increased across all subgroups defined by sex, age at cohort entry, educational attainment, and duration of follow-up. Younger individuals at cohort entry exhibited a higher excess risk (HR for those aged <20 years, 11.26; 95% CI, 3.48-36.38) compared with older individuals (HR for those aged >40 years, 2.14; 95% CI, 1.85-2.48) (*P* = .02). In the sibling-controlled cohort, similar results were observed, but they were not statistically significant. Including the first 3 years of follow-up, individuals with ILD had a higher risk compared with ILD-free individuals, and this increased risk remained even 10 years after diagnosis of ILD (HR, 2.08; 95% CI, 1.51-2.88).

**Table 3.** IR and HR of Lung Cancer Associated With ILD by Sex, Age at Cohort Entry, Educational Level, and Time Since Diagnosis (1-Year Lag Time)

Characteristics	HR (95% CI)	
	Population comparison^a^	Sibling comparison^b^
Sex		
Male	2.39 (2.01-2.84)	2.54 (1.39-4.66)
Female	1.90 (1.55-2.33)	2.68 (1.25-5.72)
*P* value for difference	.10	.92
Age at cohort entry, y		
<20	11.26 (3.48-36.38)	NA
21-40	2.10 (1.55-2.85)	3.67 (1.43-9.45)
>40	2.14 (1.85-2.48)	2.85 (1.80-4.50)
*P* value for difference	.02	.63
Educational attainment, y		
0-9	1.91 (1.56-2.35)	3.68 (1.74-7.78)
10-12	2.23 (1.83-2.72)	4.27 (1.90-9.63)
>12	2.60 (1.86-3.63)	10.40 (1.27-85.07)
*P* value for difference	.28	.66
Duration of follow-up, y^c^		
1 to <3	19.32 (6.91-53.99)	3.03 (0.52-17.82)
3 to <6	4.42 (1.91-10.21)	
6 to <10	2.88 (1.29-6.42)	
≥10	2.08 (1.51-2.88)	1.42 (0.71-2.82)
*P* value for difference	<.001	.43

Abbreviations: HR, hazard ratio; ILD, interstitial lung disease; IR, incidence rate; NA, not applicable.

^a^ Adjusted for attained age, sex, smoking-related diseases, calendar period at follow-up, and highest educational attainment.

^b^ Adjusted for attained age, sex, smoking-related diseases, calendar period at follow-up, and highest educational attainment and stratified by family identifiers.

^c^ In the population analysis, the groups are categorized as follow-up of 1 to less than 3, 3 to less than 6, 6 to less than 10, and 10 years or longer. For the sibling comparison, the groups are consolidated into 1 to less than 10 years and 10 years or longer due to the constraints of a limited sample size.

**Figure 2.**
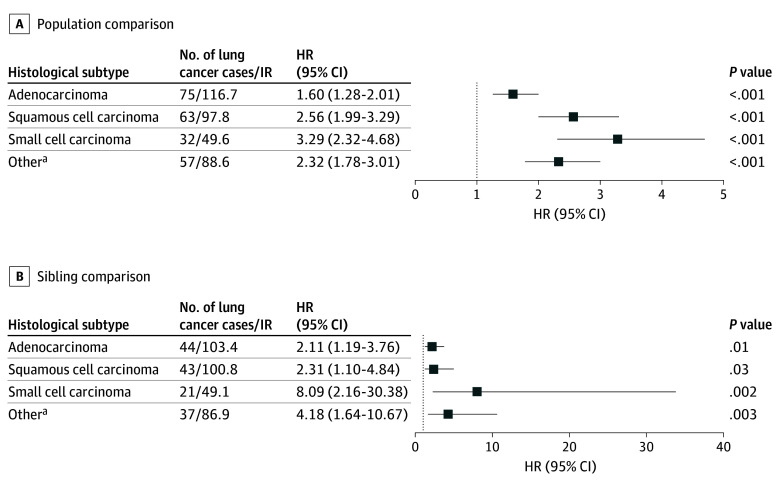
Incidence and Risk by Subtypes of Lung Cancer Associated With Interstitial Lung Disease Incidence rates (IR) per 100 000 person-years and hazard ratios (HR) with 95% CIs for various histological subtypes of lung cancer associated with interstitial lung disease (ILD) (1-year lag time). A, IRs and HRs were estimated across the general population with and without ILD. B, IRs and HRs were derived from analyses within cohorts of patients with ILD and their siblings without ILD, accounting for shared genetic and environmental factors. ^a^Includes epithelial tumor neoplasms, large cell carcinoma, undifferentiated carcinoma, bronchioloalveolar adenocarcinoma, neuroendocrine carcinoma, and adenosquamous carcinoma.

In Figure 2, we present the observed number of cases and IRs stratified by histological subtypes. We observed 75 cases of adenocarcinoma, 63 cases of squamous cell carcinoma, 32 cases of small cell carcinoma, and 57 cases of other histological subtypes. Significantly increased adjusted HRs were shown for adenocarcinoma (HR, 1.60; 95% CI, 1.28-2.01), squamous cell carcinoma (HR, 2.56; 95% CI, 1.99-3.29), small cell carcinoma (HR, 3.29; 95% CI, 2.32-4.68), and other histological subtypes (HR, 2.32; 95% CI, 1.78-3.01). Sibling-controlled analyses yielded similar results for adenocarcinoma (HR, 2.11; 95% CI, 1.19-3.76), squamous cell carcinoma (HR, 2.31; 95% CI, 1.10-4.84), small cell carcinoma (HR, 8.09; 95% CI, 2.16-30.38), and other histological subtypes (HR, 4.18; 95% CI, 1.64-10.67). Detailed results for the tumors classified as other subtypes are provided in eTable 5 in Supplement 1.

## Discussion

This study included more than 5.4 million participants with follow-up as long as 30 years and revealed that individuals diagnosed with ILD have an at least 2-fold increased risk of developing lung cancer compared with those without ILD. This association persisted for more than 10 years following the diagnosis of ILD. To account for genetic confounding, we identified individuals with ILD who had at least 1 unaffected sibling and compared those diagnosed with ILD with their unaffected siblings. The results were consistent with those observed in the general population cohort.

This study is the first, to our knowledge, to use a sibling-controlled design, thereby incorporating genetic considerations and minimizing potential familial confounding. Our findings indicate that ILD is associated with an elevated risk of lung cancer, even after adjusting for familial factors. Furthermore, additional analyses across different histological subtypes of lung cancer demonstrated that ILD increases the risk for all subtypes examined.

ILD predominantly affects the lung interstitium. However, a subset of diseases, such as alveolar proteinosis, primarily involve the alveoli but are classified under ILD due to overlapping clinical and radiological features.^6^ The potential association between ILD and lung cancer has been recognized for more than half a century. In a study among 205 patients with cryptogenic fibrosing alveolitis (CFA),^25^ patients with CFA had a 14.1-fold increased risk of developing lung cancer. Among 3712 consecutive autopsy cases, the prevalence of lung cancer in patients with usual interstitial pneumonia (UIP) was higher (48.2%) than in the age-matched general population without UIP (9.1%; *P* < .001); the prevalence of multiple primary lung cancers in patients with UIP was also markedly elevated (20.0%).^26^ In a population-based cohort study adjusted for smoking,^11^ the incidence of lung cancer was elevated 8-fold in patients with CFA. In contrast, in a study based on US death certificates,^12^ the incidence of lung cancer among patients with pulmonary fibrosis was 4.81%, lower than the 6.48% observed in the general population. However, death certificates typically record only the primary cause of death and may not capture all ILD diagnoses, which is a potential source of negative bias.^27^

Both ILD and lung cancer are associated with familial predisposition, and at least 10% of patients with IPF exhibit familial susceptibility.^28^ In genome-wide association studies, variants in host immunity, telomere maintenance, and cell division are associated with an increased risk of familial fibrotic ILD.^29^ Family history of lung cancer is the most common risk factor for lung cancer among nonsmokers in Asia.^30^

We implemented a sibling-controlled study leveraging the Swedish Multi-generation Register, which uses distinct family identifiers to connect siblings. This approach effectively mitigates genetic and familial confounders associated with lifestyle and environmental factors. When we excluded individuals with smoking-related diseases and adjusted for age, sex, calendar period at follow-up, and highest educational level, the relative risks were highly consistent across both cohorts.

Persistent stromal inflammation is thought to drive recurrent cycles of epithelial cell injury and repair in the respiratory tract, ultimately leading to cellular dysplasia. Furthermore, squamous metaplasia is more commonly observed in interstitial pneumonia associated with lung cancer, and this premalignant alteration may promote the progression to carcinoma.^31^ Dysfunction in oncogenes and tumor suppressor genes has been documented in ILD.^32^ Although various types of ILDs exhibit distinct pathophysiological mechanisms and prognoses, all forms of ILD can ultimately lead to the development of IPF.^29^ At diagnosis, pharmacological interventions are primarily aimed at improving pulmonary function and alleviating symptoms. Despite advances in treatment, these interventions do not halt or reverse the fibrotic process inherent to IPF.^33^ IPF shares numerous genetic mutations and pathway alterations with lung cancer and activates molecular pathways involved in lung cancer development.^34^ Our results indicate that although ILD increases the risk of all pathological types of lung cancer, there are variations among different types. Previous studies reported the highest relative risk of lung cancer in patients with IPF.^35,36^ We observed that small cell carcinoma exhibited the highest relative risk, followed by squamous cell carcinoma and then adenocarcinoma.

### Strengths and Limitations

Strengths of our study include its prospective, population-based, and sibling-controlled design, large size, and nearly complete capture of the entire Swedish population, as there was virtually no private inpatient care during the study period. Completeness of the Swedish Cancer Register, estimated to exceed 98%,^17^ minimizes the risk of surveillance bias. Our sensitivity analysis incorporated cases where lung cancer was recorded as the cause of death in the Cause of Death Register, which ensures that our outcome measures are comprehensive and account for any potential underreporting or misclassification of lung cancer cases. The consistency of the results across these analyses (data not shown) reinforces the robustness of our findings.

Our study also has certain limitations, most notably the lack of detailed information on smoking behavior. First, although we adjusted for smoking using proxy variables, the possibility of residual confounding cannot be entirely ruled out. Nevertheless, in sensitivity analyses restricted to individuals without any smoking-related diseases, the association between ILD and increased lung cancer risk remained and was even stronger than in the general population. To further support the robustness of this finding, we calculated the E value, which reinforced the validity of the observed association. Second, the limited number of cases restricted our ability to conduct a more detailed analysis regarding various types of ILD. Third, our study was unable to accurately attain the lesion location, preventing further analysis of the association between ILD and the anatomical site of lung cancer.

## Conclusions

In this cohort study, following adjustment for familial and other cancer-related factors, overall ILD was associated with an increased risk of various histological subtypes of lung cancer. These findings suggest that the presence of ILD should be incorporated into lung cancer risk assessment models.

## Notes

Supplement 1. **eTable 1.** Codes Used for Histological Subtype From 1958 Onward in Sweden **eTable 2.** IR and HR With 95% CI of Lung Cancer in Relation to ILD (3-Year Lag Time) **eTable 3.** HR With 95% CI of Lung Cancer in Relation to ILD Without Participants With Smoking Related Diseases **eTable 4.** Raw counts and IR by Cross-Tabulation of Smoking Related Diseases and ILD **eTable 5.** IR and HR With 95% CI for Rare Histological Subtypes of Lung Cancer Associated With ILD (1-Year Lag Time)

Supplement 2.
Data Sharing Statement
