# Supplementary material for: Interstitial Lung Disease and Risk of Lung Cancer
Source: JAMA Netw Open. 2025 Jul 9;8(7):e2519630. doi: 10.1001/jamanetworkopen.2025.19630 (PMC12242686; doi:10.1001/jamanetworkopen.2025.19630)
Supplement: Supplement 1. — eTable 1. Codes Used for Histological Subtype From 1958 Onward in Sweden eTable 2. IR and HR With 95% CI of Lung Cancer in Relation to ILD (3-Year Lag Time) eTable 3. HR With 95% CI of Lung Cancer in Relation to ILD Without Participants With Smoking Related Diseases eTable 4. Raw counts and IR by Cross-Tabulation of Smoking Related Diseases and ILD eTable 5. IR and HR With 95% CI for Rare Histological Subtypes of Lung Cancer Associated With ILD (1-Year Lag Time) [file jamanetwopen-e2519630-s001.pdf]

Supplementary Online Content

Xu H, Yin L, Bian W, Kang M, Adami H-O, Ye W. Interstitial lung disease and risk of lung cancer. *JAMA Netw Open.* 2025;8(7):e2519630. doi:10.1001/jamanetworkopen.2025.19630

eTable 1. Codes Used for Histological Subtype From 1958 Onward in Sweden
eTable 2. IR and HR With 95% CI of Lung Cancer in Relation to ILD (3-Year Lag Time)
eTable 3. HR With 95% CI of Lung Cancer in Relation to ILD Without Participants With Smoking Related Diseases
eTable 4. Raw Counts and IR by Cross-Tabulation of Smoking Related Diseases and ILD
eTable 5. IR and HR With 95% CI for Rare Histological Subtypes of Lung Cancer Associated With ILD (1-Year Lag Time)

This supplementary material has been provided by the authors to give readers additional information about their work.

© 2025 Xu H et al. *JAMA Network Open*.

eTable 1. Codes Used for Histological Subtype From 1958 Onward in Sweden

Histological subtype	PAD (1958 -)	ICDO (1993 -)
Adenocarcinoma	096	-
Squamous cell carcinoma	146	-
Small cell carcinoma	186	-
Neoplasm	-	80003
Epithelial tumor	-	80103
Large cell carcinoma	-	80123
Carcinoma, undifferentiated	-	80203
Bronchiolo-alveolar adenocarcinoma	-	82503
Neuroendocrine carcinoma	-	82463
Adenosquamous carcinoma	-	85603

© 2025 Xu H et al. *JAMA Network Open*.

eTable 2. IR and HR With 95% CI of Lung Cancer in Relation to ILD (3-Year Lag Time)

Diagnosis with ILD	Population comparison		Sibling comparison	
	No. of cases with lung cancer / IR per 100 000 person-years	HR (95%CI) [a]	No. of cases with lung cancer/ IR per 100 000 person-years	HR (95%CI) [b]
No	40672/26.2	Reference	734/87.4	Reference
Yes	147/328.0	2.02 (1.71 – 2.37)	93/309.4	2.92 (1.80 – 4.74)

Abbreviations: HR, hazard ratio; ILD, interstitial lung disease; IR, incidence rate.
[a] Adjusted for attained age, sex, smoking related diseases, calendar period at follow-up, and highest educational attainment. [b] Adjusted for attained age, sex, smoking related diseases, calendar period at follow-up, highest educational attainment and family identifiers.

© 2025 Xu H et al. *JAMA Network Open*.

eTable 3. HR With 95% CI of Lung Cancer in Relation to ILD Without Participants With Smoking Related Diseases

Without smoking related diseases	Population comparison		Sibling comparison	
	No. of cases with lung cancer / IR per 100 000 person-years	HR (95%CI) [a]	No. of cases with lung cancer / IR per 100 000 person-years	HR (95%CI) [b]
No	30707/20.3	Reference	471/64.5	Reference
Yes	117/247.9	3.68 (3.07 – 4.41)	71/225.1	3.22 (1.90 – 5.45)

Abbreviations: HR, hazard ratio; ILD, interstitial lung disease; IR, incidence rate.
[a]Adjusted for attained age, sex, calendar period at follow-up, and highest educational attainment. [b] Adjusted for attained age, sex, calendar period at follow-up, highest educational attainment and family identifiers.

© 2025 Xu H et al. *JAMA Network Open*.

eTable 4. Raw Counts and IR by Cross-Tabulation of Smoking Related Diseases and ILD

Diagnosis with ILD	With smoking related diseases (No. of cases with lung cancer / IR per 100 000 person-years)	Without smoking related diseases (No. of cases with lung cancer / IR per 100 000 person-years)
Yes	110/659.6	117/247.9
No	9885/237.1	30 707/20.3

Abbreviations: ILD, interstitial lung disease; IR, incidence rate.

© 2025 Xu H et al. *JAMA Network Open*.

eTable 5. IR and HR With 95% CI for Rare Histological Subtypes of Lung Cancer Associated With ILD (1-Year Lag Time)

Histological subtype	Population comparison		
	**No. of cases with lung cancer/ IR per 100 000 person-years**	**HR (95%CI) [a]**	***P***
Neoplasm	16/25.0	4.35 (2.63 – 7.20)	<.001
Epithelial tumor	10/15.6	2.93 (1.56 – 5.49)	<.001
Large cell carcinoma	4/6.2	1.61 (0.60 – 4.31)	.34
Carcinoma, undifferentiated	12/18.7	2.89 (1.63 – 5.12)	<.001
Bronchiolo-alveolar adenocarcinoma	6/9.4	5.57 (2.46 – 12.62)	<.001
Neuroendocrine carcinoma	1/1.6	18.84 (2.29 – 154.8)	.006
Adenosquamous carcinoma	8/12.5	2.46 (1.22 – 4.96)	.01

Abbreviations: HR, hazard ratio; ILD, interstitial lung disease; IR, incidence rate.

© 2025 Xu H et al. *JAMA Network Open*.
