# Supplementary material for: Interstitial Lung Disease and Risk of Lung Cancer
Source: JAMA Netw Open. 2025 Jul 9;8(7):e2519630. doi: 10.1001/jamanetworkopen.2025.19630 (PMC12242686; doi:10.1001/jamanetworkopen.2025.19630)
Supplement: Supplement 2. — Data Sharing Statement [file jamanetwopen-e2519630-s002.pdf]

Data Sharing Statement

Xu. Interstitial Lung Disease and Risk of Lung Cancer. *JAMA Netw Open*. Published July 09, 2025. doi:10.1001/jamanetworkopen.2025.19630

Data
Data available: No

Additional Information
Explanation for why data not available: Because the analysis was based on population-based registers in Sweden, the investigators are not in the position of owning any data that can be shared
